# Effects of graded levels of microbial fermented or enzymatically treated dried brewer’s grains on growth, digestive and nutrient transporter genes expression and cost effectiveness in broiler chickens

**DOI:** 10.1186/s12917-020-02603-0

**Published:** 2020-11-05

**Authors:** Hanan S. Al-Khalaifah, Sara. E Shahin, Anaam E. Omar, Haiam A. Mohammed, Hala. I Mahmoud, Doaa Ibrahim

**Affiliations:** 1 Environment and Life Sciences Research Center, Kuwait Institute for Scientific Research, P.O. Box:24885, 13109 Safat, Kuwait; 2 Department of Animal Wealth Development, Veterinary Economics and Farm Management, Faculty of Veterinary Medicine, Zagazig University, Zagazig, Egypt; 3 Department of Nutrition and Clinical Nutrition, Faculty of Veterinary Medicine, Zagazig University, Zagazig, 44519 Egypt; 4 Department of Physiology, Faculty of Veterinary Medicine, Zagazig University, Zagazig, Egypt; 5 Department of Animal Wealth Development, Biostatistics, Faculty of Veterinary Medicine, Zagazig University, Zagazig, Egypt

**Keywords:** Fermented dried brewer’s grain, Gene expression, Digestion gene, Nutrient transporter, Profitability

## Abstract

**Background:**

Poultry feed consists mainly of conventional grains and protein supplements, however, using treated unconventional agro-industrial by-products as replacements of corn soybean-based diet can minimize production costs and improve productivity. Therefore, in this study, the effects of fermented or enzymatically treated dried brewer grains (**DBG**) on growth, expression of digestive enzymes and nutrient transporters genes and the profitability of the rations were evaluated. A total of 1600 one-day-old Ross 308 broiler chicks were randomly distributed in 2 × 4 factorial arrangement (eight treatments with ten replicates, 20 birds/replicate). Experimental diets included two controls; negative control (basal corn-soybean diet; **NC**) and positive control (basal corn-soybean diet with exogenous enzymes; **PC**), and six diets in which basal diet was replaced by three levels of fermented DBG (**FDBG**; 5, 10 or 15%), or enzyme-treated DBG (**DBG** 5, 10 or 15%**+Enz**), for 38 days.

**Results:**

The results described that feeding FDBG (three levels) or DBG5%+Enz improved (*P <* 0.05) BW gain and feed efficiency of broilers. Also, feeding FDBG10% yielded the best improvement in weight gain (10%), compared to NC group. Increasing the inclusion levels of DBG either fermented or enzymatically treated up-regulated (*p* < 0.01) expression of digestive-genes in proventriculus (**PGC** and **PGA5**, range 1.4–1.8 fold), pancreas (**AMY2A**, **PNLIP**, **CELA1**, and **CCK**; range 1.2–2.3 fold) and duodenum (**CAT1**, **CAT2**, **GLUT1**, **GLUT2**, **LAT1**, **Pep1**; range 1.3-3 fold) when compared to NC group. Feeding treated DBG significantly increased (*p* < 0.05, range 4.5–13.6%) gizzard relative weight compared to NC and PC groups. An additional benefit was lower (*p *< 0.01) cholesterol content from 66.9 mg/100 mg (NC) to 62.8 mg/100 mg (FDBG5 or 10%) in thigh meat. Furthermore, the least cost feed/kg body gain was achieved in FDBG10% and DBG5%+Enz groups, with approx. 16% reduction compared to NC cost, leading to increasing the income gross margin by 47% and 40% in FDBG10% and DBG5%+Enz groups, respectively.

**Conclusions:**

Substitution of corn-soybean based diet with 10% FDBG or 5% DBG+Enz resulted in better growth and higher economic efficiency of broilers chickens.

## Background

In commercial poultry, the feeding costs of broilers contribute up to 70% of the total production costs. And, since, global feed prices are increasing, it is important to explore alternative or unconventional feed ingredients to achieve cost-effective poultry production [1]. However, the high fiber and low protein contents and presence of antinutritional factors (**ANF**) in unconventional feed represent a critical challenge for their use in poultry feed. One of the most prominent plant by-products arise mainly from oilseed mills and brewing industries [2]; called dried brewer’s grains (**DBG**). The latter could be an appropriate low-cost replacer for traditional feedstuffs (e.g., corn and soybean meal), as it contains a fair amount of feed residues following the brewing process [3–6]. It consists of around 20% crude protein, 6% ether extract, 15% crude fiber and 4% ash, in addition it has an adequate amount of essential AA (0.4% methionine, 0.9% lysine, 1.2% phenylalanine, 0.4% tryptophan, 1.1% threonine and 1.6% valine) [7].

However, the use of DBG in poultry feed has certain limitations such as elevated moisture content, non-starch polysaccharides (**NSP**) and tannins hindering feed utilization and inhibiting the digestive enzymes and nutrients absorption [8]. Adding different exogenous enzymes is one option to mitigate ANF and improve DBG utilization, in addition to supporting bird’s endogenous enzymes and nutrient absorption thus improving digestibility and availability of nutrients [9, 10]. However, supplementation of exogenous enzymes in poultry feed still have limitations which reduce their beneficial effects, such as the wide range of pH along bird’s GIT (while their optimum pH is between 4 and 6), the shorter time for digesta retention in proximal GIT (their main site of action) and the possibility of hydrolysis by endogenous proteolytic enzymes [11].

The other possible strategy for improving DBG quality in broiler diet is through the fermentation approach. Fermentation is a dynamic process containing microorganisms, substrates and environmental conditions to transform complex substrates into simpler ones [12]. As the fermentation intensity depends on the number of microorganisms, thus adding fungi or bacterial strains can be a favorable aid during fermentation. It is known that fermentation improves microbial and nutritional quality of feedstuffs by increasing protein and lipids and lowering fiber content [13]. As well as, improving the availability of vitamins and AAs [14, 15] and reducing several ANF and toxic compounds [16]. Fermentation has also been proven to enhance nutrient digestibility of organic matter, fiber and calcium [17], and the palatability of feedstuff [18]. In addition, Tan et al. [19] confirmed an enhanced nutritional content of fermented DBG through biological fermentation by adding certain fungi and bacteria.

The nature of broiler’s diet can modulate the expression of important genes associated with nutrient transport and digestive enzymes [20, 21]. In fact, improving nutrient transport, through the up-regulation of the transporter encoding genes, lead to accelerated nutrient entry into the intestinal epithelium and then to the circulation [22]. It is identified that transporters of glucose (**GLUT1** and **GLUT2**), cationic amino acid (**CAT1** and **CAT2**) and peptide (**PepT1** and **PepT2**) in the intestinal epithelium are associated with the capacity of nutrient absorption [21, 23–25].

The effects of different DBG treatments (fermentation or added enzymes) on expression of genes controlling digestive enzymes and nutrients transporters in broilers remains not fully understood. In addition, the economic efficiency of replacing costly conventional feed ingredients should promote the use of low-priced unconventional fermented feedstuffs as DBG in broiler diets. Therefore, the present study investigated the impact of feeding different levels of DBG either fermented or enzyme-treated on the gene expression of selected digestive enzymes and nutrient transporters, growth performance and economic efficiency of broiler chickens.

## Results

### Growth indices

The highest body weight and weight gain (*P* < 0.05) were observed in FDBG10% group followed by FDBG5% and DBG5%+Enz groups, Table 1. The lowest total feed intake (*P* < 0.05) was observed in group fed FDBG15% followed by groups fed on DBG10%+Enz and negative control. The feed conversion ratio (FCR) was improved (*P* < 0.05) in groups fed FDBG5%, FDBG10% and DBG5%+Enz when compared with NC and PC groups.

**Table 1:** Effects of different levels of fermented or enzymatically treated dried brewers’ grains on growth performance and carcass traits of broilers^1^

Treatmnets^2^	NC	PC	FDBG15%	FDBG10%	FDBG15%	DBG5%+Enz	DBG10%+Enz	DBG15%+Enz	SEM	*P*-value
Allover growth performance parameters^3^										
BW, g/bird	2414^e^	2559^c^	2587^b^	2690^a^	2432^d^	2593^b^	2332^f^	2188^g^	9.26	< 0.001
BWG, g/bird	2369^e^	2514^c^	2542^b^	2645^a^	2387^d^	2549^b^	2287^f^	2143^g^	9.20	< 0.001
FI, g/bird	3939^d^	4109^a^	4052^b^	4001^c^	3883^e^	3993^c^	3915^de^	4067^b^	59.82	< 0.001
FCR	1.66^c^	1.63^cd^	1.57^e^	1.51^f^	1.63^d^	1.57^e^	1.71^b^	1.90^a^	< 0.006	< 0.001
Carcass traits^4^										
Dressed weight	1458^c^	1576^b^	1593^b^	1641^a^	1547^b^	1603^a^	1422^c^	1321^c^	0.75	< 0.001
Liver, %	2.52	2.36	2.5	2.32	2.48	2.63	2.26	2.61	0.02	0.11
Gizzard, %	2.20^c^	2.26^bc^	2.31^b^	2.49^a^	2.50^a^	2.36^b^	2.40^ab^	2.43^a^	0.10	0.03
Spleen, %	0.17	0.14	0.15	0.14	0.15	0.14	0.13	0.14	0.01	0.27
Thymus, %	0.34	0.31	0.38	0.34	0.34	0.37	0.37	0.33	0.05	0.08
Bursa, %	0.06	0.67	0.07	0.08	0.07	0.07	0.08	0.09	0.01	0.5
Abdominal fat, %	1.69	1.72	1.70	1.74	1.74	1.72	1.78	1.76	0.30	<0.32

^a-g^Means within the same row carrying different superscripts are significantly different at *P* < 0.05. ^1^Data represent the mean value of ten replicate pens of 20 birds. ^2^Treatments include: NC (negative control), PC (positive control), FDBG5% (5% fermented dried brewers grains), FDBG10% (10% fermented dried brewer grain), FDBG15% (15% fermented dried brewers grains), DBG5%+Enz (5%dried brewers grains mixed with enzymes), DBG10%+Enz (10% dried brewers grains mixed with enzymes), DBG15%+Enz (15% dried brewers grains mixed with enzymes).^3^*BW* Body weight, *BWG* Body weight gain, *FI* Feed intake, *FCR* Feed conversion ratio.^4^Carcass traits (*n*=5/treatment)

### Carcass traits

The highest dressing percentage (*P* < 0.05) was observed in groups fed FDBG10% and DBG5%+Enz, Table 1. Feeding on DBG either fermented or enzymatically treated significantly increased (*P* < 0.05) gizzard relative weight. Feeding of broiler chicks on treated DBG had no effect on the relative weights of liver, spleen, thymus, bursa and abdominal fat.

### Nutrient transporter and digestive enzyme genes expression

#### Proventriculus pepsinogen (PGC and PGA5) genes expression (Fig. 1)

The upregulation of proventriculus pepsinogen (PGC and PGA5) genes was more prominent (*P* < 0.01) in groups with higher levels of DBG either fermented or enzymatically treated when compared with the NC and PC groups.

**Fig. 1:**
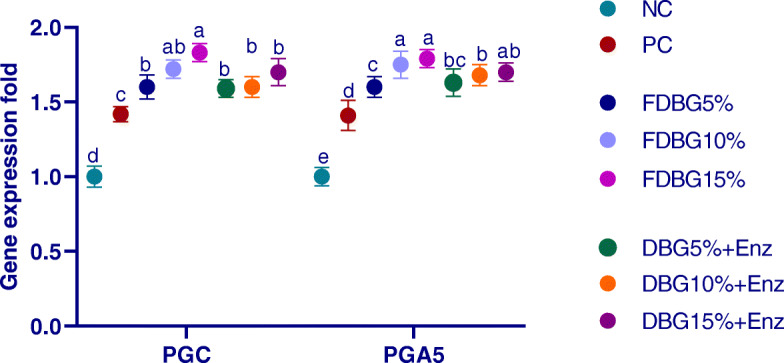
Effects of different level of fermented or enzymatically treated dried brewers’ grains on expression of proventriculus gene (PGC and PGA5). ^a-c^ Means within the same column carrying different superscripts are significantly different at *P* < 0.05

#### Pancreatic genes expression, AMY2A and PNLIP (Fig. 2a), CELA1 and CCK (Fig. 2b)

The mRNA expression of pancreatic AMY2A, PNLIP and CELA1 was upregulated (*p* < 0.01) in response to all inclusion levels of treated DBG. The higher expression of AMY2A gene was more prominent in groups fed higher levels of FDBG. Regarding CCK expression level, groups fed FDBG10% and 15% had the highest expression level while FDBG5% and enzymatically treated DBG groups had the intermediate expression levels and control groups had the lowest expression level.

**Fig. 2:**
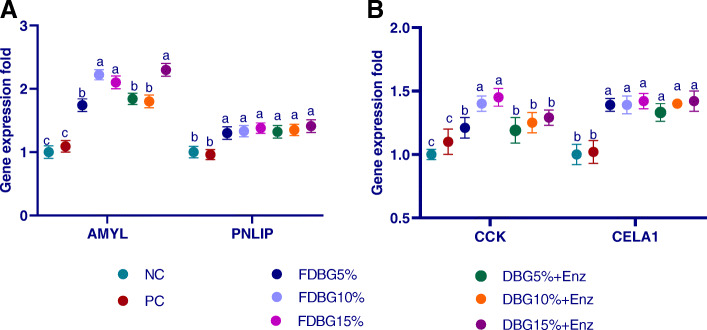
Effects of different level of fermented or enzymatically treated dried brewers’ grains on expression of pancreatic genes (AMY2A, PNLIP; (**a**), CCK and CELA1; (**b**). ^a-c^Means within the same column carrying different superscripts are significantly different at *P* < 0.05

#### Duodenal genes expression, GLUT1 and GLUT2, (Fig. 3a); PepT1 and PepT2 (Fig. 3b) and CAT1, CAT2 (Fig. 4a) and LAT1 (Fig. 4b)

The mRNA expression of GLUT1, GLUT2, were up regulated in response to the dietary inclusion of treated DBG. The higher levels of Broilers fed on Fermented or enzymatically treated DBG or PC significantly up-regulated (*P* < 0.01) the CAT1, CAT2, while PepT2 expression not affected by feeding on treated DBG (*P* > 0.05). Increasing inclusion of fermented or enzymatically treated DBG significantly up-regulated (*P* < 0.01) LAT1 gene expression. Moreover, the effect of fermentation on upregulation of previous genes was more prominent than enzymes treatment. Increasing the inclusion levels from DBG either fermented or enzymatically treated up-regulated (*P*  < 0.05) the expression of GLUT1, GLUT2 genes than in control positive group.

**Fig. 3:**
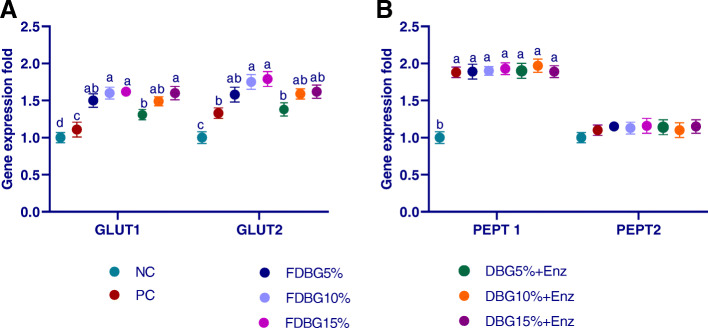
Effects of different level of fermented or enzymatically treated dried brewers’ grains on expression of duodenal genes, GLUT1, GLUT2; (**a**) and PEPT1 and PEPT2; (**b**). ^a-b^Means within the same column carrying different superscripts are significantly different at *P* < 0.05

**Fig. 4:**
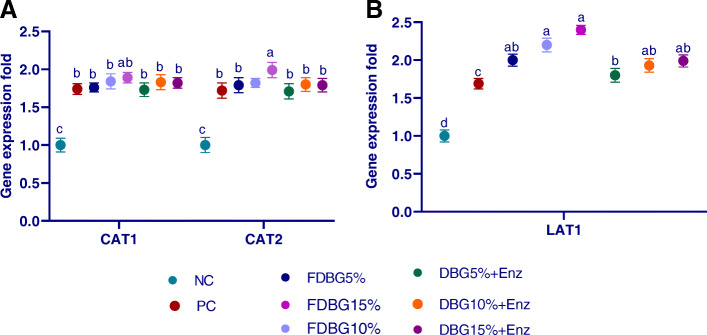
Effects of different level of fermented or enzymatically treated dried brewers’ grains on expression of duodenal genes (CAT1, CAT2; (**a**) and LAT1; (**b**)). ^a-c^Means within the same column carrying different superscripts are significantly different at *P* < 0.05

##### Chemical composition of meat

The moisture, crude protein and cholesterol contents of breast meat were not different among all treatments, Table 2. However, the groups fed on FDBG5% and FDBG10% had the lowest cholesterol content (*P* < 0.05) in thigh meat

**Table 2:** Effects of different levels of fermented or enzymatically treated dried brewers’ grains on chemical composition of meat

Treatments^1^	Breast moisture %	Breast protein %	Breast cholesterol (mg/100mg)	Thigh cholesterol (mg/100mg)
NC	70.60	21.97	62.36	66.91^ab^
PC	70.81	22.23	62.25	66.85^ab^
FDBG5%	70.93	22.80	61.46	62.84^d^
FDBG10%	70.64	22.12	61.54	62.82^d^
FDBG15%	70.56	23.00	61.34	64.33^c^
DBG5%+Enz	71.00	22.94	61.83	65.67^bc^
DBG10%+Enz	70.63	22.49	61.97	68.00^a^
DBG15%+Enz	70.27	22.10	61.73	68.70^a^
SEM	0.06	0.07	0.30	0.04
*P*-value	0.09	<0.08	<0.07	<0.001

^a-c^Means within the same column carrying different superscripts are significantly different at *P* < 0.05, n=5/treatment). ^1^Treatments include: NC (negative control), PC (positive control), FDBG5% (5% fermented dried brewers grains), FDBG10% (10% fermented dried brewer grain), FDBG15% (15% fermented dried brewers grains), DBG5%+Enz (5%dried brewers grains mixed with enzymes), DBG10%+ Enz (10% dried brewers grains mixed with enzymes), DBG15%+Enz (15% dried brewers grains mixed with enzymes)

.

##### Serum biochemical parameters

The concentrations of total protein, albumin, globulin, triglyceride, ALT and AST were not affected (*P* > 0 0.05) by any of the experimental diets, Table 3. However, the serum concentrations of total cholesterol and LDL were significantly lowered (*P *< 0.05) in broilers fed FDBG when compared with other groups.

**Table 3:** Effects of different levels of fermented or enzymatically treated dried brewers’ grains on some serum biochemical parameters

Treatments^1^	Total protein (g/dl)	Albumin (g/dl)	Globulin (g/dl)	TC (mg/dl)	TAG (mg/dl)	HDL (mg/dl)	LDL (mg/dl)	AST (U/L)	ALT (U/L)
NC	4.55	3.14	1.41	174.50^a^	72.13	77.41	82.75^a^	1.63	51.38
PC	4.87	3.28	1.59	165.16^a^	77.22	76.03	73.68^a^	1.51	51.30
FDBG5%	5.19	3.75	1.440	154.52^b^	75.67	76.72	62.66^b^	1.51	51.83
FDBG10%	5.13	3.07	2.06	156.94^b^	72.27	78.36	64.13^b^	1.64	51.67
FDBG15%	6.02	3.03	2.99	157.33^b^	76.38	78.30	63.75^b^	1.60	53.18
DBG5%+Enz	5.09	3.32	1.77	162.80^ab^	79.14	73.21	73.75^ab^	1.61	52.61
DBG10%+Enz	4.92	3.583	1.54	172.95^a^	75.81	69.97	87.83^a^	1.63	52.86
DBG15%+Enz	4.88	3.61	1.31	171.10^a^	74.84	71.07	85.06^a^	1.66	50.76
SEM	0.03	0.17	0.07	4.542	15.01	3.13	11.43	0.60	0.001
*P*-value	0.06	0.91	0.13	<0.001	0.972	0.119	<0.003	0.71	0.06

^a-b^Means within the same column carrying different superscripts are significantly different at *P* < 0.05.^1^Treatments include: NC (negative control), PC (positive control), FDBG5% (5% fermented dried brewers grains), FDBG10% (10% fermented dried brewer grain), FDBG15% (15% fermented dried brewers grains), DBG5%+Enz (5%dried brewers grains mixed with enzymes), DBG10%+ Enz (10% dried brewers grains mixed with enzymes), DBG15%+Enz (15% dried brewers grains mixed with enzymes). TC (Total Cholesterol), TAG (Triglyceride), HDL (high density conjugated protein), LDL (low density protein), AST (Aspartate aminotransferase), ALT (alanine aminotransferase)

##### Economic indices

Total feed cost and total cost decreased significantly (*P *< 0.05) with increasing DBG levels, either fermented or mixed with enzymes, Table 4. The highest values of total expenses were recorded in NC (2.71$) and PC (2.80$) groups. While the highest gross margin (*P* < 0.05) was evident in broilers fed FDBG10% (1.58$) followed by DBG5%+Enz (1.50$). The lowest cost/kg BW gain of broilers and highest cost benefit ratio were achieved in groups fed FDBG10% and DBG5%+Enz.

**Table 4:** Effects of different level of fermented or enzymatically treated dried brewers’ grains on the economic indices

Treatments^1^	Feed cost^2^	Total expenses	Total revenue	Gross margin	benefit cost ratio	Cost/ kg BW gain
NC	1.61^b^	2.71^b^	3.79^c^	1.07^g^	0.66^e^	0.69^a^
PC	1.70^a^	2.80^a^	4.01^b^	1.21^e^	0.70^d^	0.68^a^
FDBG5%	1.57^c^	2.67^c^	4.05^b^	1.38^c^	0.87^b^	0.61^bc^
FDBG10%	1.52^d^	2.62^d^	4.21^a^	1.58^a^	1.04^a^	0.58^d^
FDBG15%	1.44^f^	2.53^f^	3.80^c^	1.28^d^	0.89^b^	0.60^c^
DBG5%+Enz	1.46^e^	2.56^e^	4.06^b^	1.50^b^	1.02^a^	0.56^d^
DBG10%+Enz	1.42^g^	2.56^e^	3.66^d^	1.14^f^	0.80^c^	0.62^b^
DBG15%+Enz	1.46^e^	2.52^g^	3.44^e^	0.88^h^	0.60^f^	0.68^a^
SEM	2.59	2.59	0.003	0.006	0.006	1.15
*P*-value	<0.001	<0.001	<0.001	<0.001	<0.001	<0.001

^a-h^Means within the same column carrying different superscripts are significantly different at *P* < 0.05. ^1^Treatments include: NC (negative control), PC (positive control), FDBG5% (5% fermented dried brewers grains), FDBG10% (10% fermented dried brewer grain), FDBG15% (15% fermented dried brewers grains), DBG5%+Enz (5%dried brewers grains mixed with enzymes), DBG10%+ Enz (10% dried brewers grains mixed with enzymes), DBG15%+Enz (15% dried brewers grains mixed with enzymes).^2^Cost/kg diet$ = NC (0.411) , PC (0.415), FDBG5% (0.388), FDBG10% (0.381), FDBG15% (0.370), DBG5%+Enz (0.367), DBG10%+Enz (0.362), DBG15% +Enz (0.359). price/kg meat = $ 1.52. fixed costs = $ 1.1

## Discussion

Microbial fermentation is a recent low-cost method to enhance the nutritional value of unconventional feed ingredients for broiler chickens. Also, there is a growing interest in the introduction of fermented feed to broiler diets due to its positive impacts on the gut health and growth performance [26, 27]. Thus, subjecting unconventional feed as DBG to microbial fermentation may be a sound alternate to improve DBG nutritive value. In the current study, microbial fermentation improved nutritive value of DBG which allowed its inclusion up to 10% in the formulated diet and led to better broiler’s performance. Similar studies have reported that microbial fermentation is an effective method to degrade ANFs and provide probiotics in feed, thus enhancing the nutritional quality and improving animal growth [28–30]. Improved nutritional quality due to fermentation process is through increasing crude protein and reducing crude fiber contents [29].

Therefore, increased protein content in fermented DBG may arise from (1) synthesis of microbial proteins and enzymes during fermentation [31], (2) increasing microbial population which is composed mainly from protein [32], and (3) enhancing the activities of proteolytic microorganisms which causes an increase in free amino acids and peptides in fermented products [33]. Also, fiber reduction after fermentation is due to increased microbial population which produce fiber degrading enzymes [34]. Also, addition of *B. subtilis* during fermentation enhanced growth and proliferation of lactic acid bacteria (**LAB**) and in turn reduced pH thus inhibited the growth of pathogenic bacteria [30]. Moreover, during anerobic fermentation process, the addition of *S. cerevisiae* was preferred due to its greater pH lowering capacity [35] and enzyme producing ability as β -glucanase, phytase and invertase [36–38].

The improved BW and FCR of broiler chickens during the whole grow-out period (d 1–38) in groups fed FDBG (up to 10%), is a reflection to the improved nutritional properties of FDBG compared to enzymatically treated DBG. Similarly, recent reports showed that the fermentation help in the production of functional feeds that can improve the microecology and health of broiler’s gut and their productive performance [39]. In addition, fermentation was associated with a high concentration of organic acids which increased the number of LAB leading to lowered gastric pH and pathogenic microbial activity [17, 40, 41]. All these previous characteristics of fermented feed should prevent pathogenic feed contamination prior to feeding [42], protect chicken gastrointestinal health [43, 44] and improve their growth [45].

Adding selected microbial strains as *B.subtilis, L. rhamnosus and S. cerevisiae* during fermentation of DBG not only improved DBG nutritional value but also enhanced birds growth rate. In accordance with our results, microbial fermentation with *B. Subtilis* can increase feed palatability [46], secrete digestive enzymes (proteases, lipases and amylases), stimulate good digestion and absorption of nutrients, and, produce active substances (bacitracin, polymyxin, nystatin, gramicidin) which inhibit endogenous pathogens [47]. The last authors also suggested that microbial phytase and protease enzymes may be accountable for decreasing the contents of phytic acid and allergic proteins, respectively. In addition, *S. cerevisiae* effects were attributed to maintaining beneficial microbial population and modifying metabolism by enhancing the activity of digestive enzymes [48].

Treating DBG with enzymes (especially the DBG5%+ENZ group) also had positive effects on improving broiler’s FCR agreeing with previous reports describing that inclusion of exogenous enzymes in conventional or unconventional broiler’s diet led to lower digesta viscosity, countered anti-nutritional factors and assisted in the development of important microbiota [49–51]. Also, the incorporation of multienzyme (xylanase, amylase, and protease) in broiler’s diet improved fibers utilization [52]. While, supplementing β-glucanase to barley-based diet reduced ileal viscosity and altered the concentration of short chain fatty acids in the crop and ceca thus increased broilers body weight gain [53, 54].

Increased gizzard weight observed in all DBG groups may be due to the presence of NSP in DBG, which was shown to beneficially affect nutrient utilization by (1) increased proventriculus and gizzard activity, (2) triggered hydrochloric acid and digestive enzyme secretion and (3) augmented hind gut bacterial fermentation [55]. Moreover, a well-developed gizzard musculature can produce strong reverse peristalsis contractions and increase the digesta refluxes in digestive tract thus re‐exposing the digesta to hydrochloric acid and pepsin and help their mixing [56–58].

The current study demonstrated that genes essential for digestion and nutrient transport were upregulated in the proventriculus, duodenum and pancreas of broilers in line with increased level of structural fiber components (by increasing DBG level; either fermented or enzymatically treated) in broiler’s diet. This effect was more prominent with FDBG than in enzymatically treated DBG. In accordance with the upregulated pepsinogen expression after feeding treated DBG in our study, it was shown that feeding on high fiber diet upregulated the expression of pepsinogen A and C in birds [55]. In addition, the high fiber content in DBG should stimulate frequent and powerful contractions of the gizzard and relocating the digesta back into the proventriculus leading to more stimulation of proventricular function, as previously reported in high fiber diets [55]. Additionally, the upregulation of pepsinogen A and C expression with higher inclusion levels of FDBG may be related to increased LAB, more lactic acid and lower pH, which accelerated the proliferation of gut epithelial cells [59, 60] and stimulated gastric chief cells to release pepsinogen [61]. While, feeding enzymatically treated DBG could enhance pepsinogen via the supplied exogenous enzymes which upregulated the expression of digestive enzymes [62]. So, in our study increasing gizzard contraction and pepsin production in proventriculus can result in a more active foregut and better nutrient digestion.

Also, in this study, the nature of diet affected the magnitude of pancreatic secretion, as high dietary carbohydrates or fat stimulated pancreatic secretions and increased amylase and lipase concentration in serum [63–65]. In the current study, the increased expression of pancreatic enzymes-related genes by using DBG was in accordance with previous reports which stated that insoluble fiber can increase pancreatic enzymes such as chymotrypsin [66, 67]. Similarly, oat hulls with high fiber content stimulated the secretion of pancreatic amylase and thus increased amylase activity in the jejunum [68]. Moreover, the activities of pancreatic α-amylase and lipase in broiler chickens were significantly increased by feeding wheat and barley-based diet compared to corn-based diet with enzymes supplementation [69]. Also, elevated CCK expression with increased levels of treated DBG was related to improved gut motility, gastro-duodenal reflux and secretion of pancreatic enzymes which in turn increased CCK release and improved the digestibility [70, 71]. Likewise, fermented feeds elevated pancreatic AMY2A and CCK expression which in turn increased the secretion of pancreatic amylase and cholecystokinin in broilers [44, 72]. Additionally, the upregulated expression of pancreatic lipase in all treated DBG groups led to higher lipase secretion and resulted in better fat absorption [73]. Furthermore, the probiotic properties of FDBG led to improving intestinal structure, absorptive surface area and expression of brush border enzymes, thus achieving boosted digestion and absorption [74, 75]. Equally, *B. subtilis* based-diet up-regulated digestive genes such as pancreatic lipase, carboxypeptidase and chymotrypsin-like elastase in the gut [76].

Nutrient absorption, in the small intestine, is mediated by transporter proteins expressed in enterocytes. Up-regulation of these transporters improved nutrient transport capability and accelerated nutrients influx into the intestinal epithelial cells and then to all body (Ruhnke et al., 2015). In our study, GLUT1, GLUT2, CAT1, CAT2 and PEPT1 were all upregulated after feeding on DBG (either fermented or enzymatically treated). In agreement with our results, the addition of exogenous dietary enzyme for broilers significantly upregulated the expression of intestinal PEPT1 and GLUT2 thus facilitated micronutrients absorption [62]. Similarly, inclusion of fiber-rich sugarcane bagasse upregulated CAT1, LAT2 and PepT2, which facilitated the bidirectional transfer of cationic amino acids, Na^+^−dependent neutral/cationic amino acid exchange, and di- and tri-peptides transport in intestine, respectively [55]. The increase in carbohydrates consumption led to higher expression levels of glucose transmitters which increased glucose absorption [77]. Also, the expression of GLUT2 and PEPT1 genes was upregulated after supplementation of xylanase which may indicate an improved absorption in birds [78, 79]. All this confirms that increased entry of nutrients from the intestinal lumen into the enterocyte resulted in improved tissue protein synthesis and enhanced feed utilization as previously highlighted [80].

No considerable changes were noticed in the activities of liver metabolic enzymes (ALT and AST) among different experimental groups indicating absence of any metabolic side effects. Additionally, there was no substantial impact of treated DBG on any of the serum constituents (Albumin, globulin, cholesterol, triglyceride and high-density lipoprotein), except for total cholesterol and low-density lipoprotein LDL which were decreased in FDBG groups compared to control or enzymatically treated groups. This FDBG-caused reduction in blood cholesterol levels could be through, (1) inhibiting enzymes involved in biosynthesis of cholesterol (as 3-hydroxy-3-methylglutaryl CoA reductase) (2) increasing bile acid production, or (3) decreasing cholesterol synthesis and absorption in the gastrointestinal tract [81]. In addition, fermentation-related LAB decreased Niemann-Pick C1-like 1 expression levels in enterocytes leading to less cholesterol absorption [82].

In the present study, reduced cholesterol levels in blood was also reflected in lower cholesterol contents in thigh meat, following FDBG, which agree with previous findings [83]. In addition, it may be attributed to increased total fecal excretion of cholesterol via the bile [84]. Also, similar results were found when cholesterol concentration in breast and thigh meat decreased in turkey fed on fermented feed by-products [85]. Likewise, using of 10% fermented feed in broiler diet significantly decreased fat content in breast muscle [86].

It is accepted that using of non-conventional dietary sources can reduce the feeding cost of broilers which is a main constraint for profit in broiler industry. As described in our study the feed cost and total costs were higher in control groups, which is clearly due to the costs of conventional ingredients. However, feed cost and cost/kg diet were decreased with increasing the level of DBG in the rations, this is mainly due to its lower purchase price compared to conventional ingredients in the control diet. This was reflected in better net profit and total return values in 10% FDBG and 5% DBG + enzymes groups, which showed a decrease in feed cost/kg together with better feed utilization and body weight gain compared to the remaining groups. Similarly, appreciable financial gain was reported after increasing the inclusion level of exogenous enzymes fortified dried brewer grain in the broilers diet [87]. Also, combining exogenous enzymes with non-conventional ingredients is a valid practice to reduce the cost of feeding and to allow better utilization of the non-conventional feed ingredients that are typically rich in fibers and are difficult to be utilized by poultry endogenous enzymes. This was achieved in this study in the group fed on enzymatically treated DBG at 5% inclusion level. Additionally, previous findings indicated that the total cost of production and feed cost/kg decreased with increasing BDGs level [88]. Moreover, the feed cost effectiveness in FDBG 10%, agreed with previous reports [89, 90] which reported that probiotic produced by solid substrate fermentation (SSF) is economically and environment-friendly. Furthermore, we confirm previous hypothesis that using fermented feed containing probiotics would be of economic value via improving the broiler feed conversion ratio [91]. Therefore, from an economic point of view, it is clear in our results that fermentation of DBG achieved better economic production than enzymatically treated DBG.

## Conclusions

Introduction of new nutritional strategies (such as microbial fermentation) for broilers, lead to better utilization of unconventional feed as DBG and improving the revenue. Fermentation of DBG positively affected the expression of digestive and nutrient transporters genes which regulate nutrients and energy availability necessary for optimum bird-growth, more than enzymatic treated DBG. These outcomes should encourage the poultry feed industry to apply cost effective protocols by incorporating fermented unconventional products in broiler diets.

## Methods

### Microbial fermentation of Dried Brewer Grains

The dried brewer’s grains (DBG) were obtained from beer factory and dried for 12 h at 40 °C then finely ground, then were fermented by different microbes. *Bacillus subtilis* (NCIMB 15,204) was activated with nutrient broth (5 ml) at 37 °C for 24 h. *Lactobacillus rhamnosus* (ATCC 7469) cultures were anaerobically incubated in De Man, Rogosa and Sharpe (MRS) (Oxoid Ltd., Basingstoke, Hampshire, UK) at 37 °C for 24 h for activation. *Saccharomyces cerevisae* (CGMCC No. 2.1793) was activated by incubation at 37 °C for 48 h in glycerol and MRS broth. A mixed liquid culture of approximately 1 × 10^6^ CFU/ml of each *S. cerevisiae, L. rhamnosus* and *B. subtilis*, were utilized by the same ratio and mixed with DBG. The mixture was packaged and sealed in polyethylene bags with a one-way valve to permit carbon dioxide release during fermentation. Through the initial phase of fermentation, *S. cerevisae* consumed the oxygen in the bag and generated significant amount of carbon dioxide. This process produced an anaerobic and acidic conditions which enabled both *L. rhamnosus* and *B. subtilis* to grow and germinate. The fermentation was held for 25 days. Samples of dried brewers’ grains (DBG) were collected for chemical analysis according to AOAC [92], and shown in Table 5.

**Table 5:** Chemical analysis (% on DM basis) of unfermented (UFDBG) and fermented (FDBG) dried brewers’ grains

Constituent (%)	^1^UFDBG	^2^FDBG
Crude protein	28.20±0.15^b^	29.60±0.10^a^
Ether extract	6.20±0.20^b^	6.90±0.16^a^
Crude fiber	12.60±0.19^a^	10.80±0.17^b^
^3^NDF	54.66±0.08^a^	51.36±0.07^b^
^4^ADF	20.36±0.09^a^	18.46±0.20^b^
Lignin	5.26±0.10^a^	4.00±0.20^b^

^a-b^Means within the same row carrying different superscripts are significantly different at *P* < 0.05. ^1^UDBG (un-fermented dried brewer grains), ^2^FDBG (fermented dried brewer grains).^3^NDF (Neutral detergent fiber), ^4^ADF Acid detergent fiber. Values are expressed as means± standard error

### Birds, experimental design, and management

One-day-old male Ross 308 broiler chicks (n = 1600) were obtained from a commercial chick producer (Dakahlia Poultry, Mansora, Egypt) and weighted and randomly assigned to one of the eight experimental treatments (ten replicates/treatment, and 20 birds/replicate) in 2 × 4 factorial arrangement. Treatments consist of negative control (**NC**) birds received corn-soybean based diet; positive control (**PC**) birds received corn-soybean based diet with exogenous enzymes and in the remaining six diets, the basal diet was replaced by three levels of fermented DBG (**FDBG**; 5, 10 or 15%), or enzyme-treated DBG (**DBG** 5, 10 or 15%**+Enz**). A commercial multi-exogenous enzyme was added was added to **PC** and enzyme treated DBG diets at a concentration of 1 gm/kg diet.

The birds were raised in a naturally ventilated open house with sawdust as litter. The feed and water were available ad-libitum and the broilers were raised in floor pens over a period of 38 days. The temperature, relative humidity and lighting were adjusted following Ross 308 management guidelines [93]. All diets were offered in mash form and the basal diets were formulated (starter, grower-finisher) according to nutrition specification of Ross broiler handbook [93], Tables 6 and 7. The chemical analyses (moisture, crude protein, ether extract and crude fiber) of all feed ingredients were conducted according to AOAC [92]. A commercial multi-exogenous enzymes (KEMZYME® Plus Europa NV, Belgium), containing Xylanase, B-glucanase, cellulase, alpha-amylase and protease. After the study, all remaining birds were released.

**Table 6:** The ingredients and nutrient level of diets during starter stage

Diet composition (%)	Experimental diets^a^							
	NC	PC^b^	FDBG 5%	FDBG 10%	FDBG 15%	DBG5% +Enz^b^	DBG10% +Enz^b^	DBG15% +Enz^b^
Yellow corn	57.50	57.50	55.25	53.00	50.50	54.55	51.30	48.45
Soybean meal	31.80	31.80	29.00	26.20	23.70	29.40	27.20	24.70
Corn gluten	3.30	3.30	3.30	3.30	3.30	3.30	3.30	3.30
FDBG^c^	0	0	5.00	10.00	15.00	0	0	0
DBG^d^	0	0	0	0	0	5.00	10.00	15.00
Soybean oil	3.00	3.00	3.00	3.00	3.00	3.30	3.70	4.00
Calcium carbonate	1.00	1.00	1.00	1.00	1.00	1.00	1.00	1.00
Calcium diphasic phosphate	1.50	1.50	1.50	1.50	1.50	1.50	1.50	1.50
Common salt	0.30	0.30	0.30	0.30	0.30	0.30	0.30	0.30
Premix^e^	0.90	0.90	0.90	0.90	0.90	0.90	0.90	0.90
L-Lysine	0.20	0.40	0.25	0.30	0.30	0.25	0.30	0.35
DL-Methionine	0.20	0.20	0.20	0.20	0.20	0.20	0.20	0.20
Choline chloride	0.20	0.20	0.20	0.20	0.20	0.20	0.20	0.20
Anti-mycotoxin	0.10	0.10	0.10	0.10	0.10	0.10	0.10	0.10
Calculated composition								
ME (Kcal/Kg)	3105	3105	3105	3105	3101	3102	3103	3101
CP (%)	22.50	22.50	22.50	22.50	22.50	22.50	22.50	22.50
EE %	5.44	5.44	5.67	5.90	6.10	5.89	6.44	6.90
CF (%)	2.56	2.56	2.94	3.30	3.32	3.12	3.68	4.23
Calcium (%)	1.11	1.11	1.10	1.10	1.11	1.11	1.10	1.10
Available phosphorous (%)	0.51	0.51	0.49	0.47	0.46	0.49	0.48	0.46
Lysine (%)	1.27	1.27	1.27	1.28	1.26	1.27	1.28	1.28
Methionine (%)	0.57	0.57	0.58	0.58	0.59	0.58	0.59	0.60

^a^NC (negative control), PC (positive control), FDBG5% (5% fermented dried brewers grains), FDBG10% (10% fermented dried brewer grain), FDBG15% (15% fermented dried brewers grains), DBG5%+Enz (5%dried brewers grains mixed with enzymes), DBG10%+Enz (10% dried brewers grains mixed with enzymes), DBG15%+Enz (15% dried brewers grains mixed with enzymes). ^b^Commercial multi-exogenous enzyme was added was added to PC and enzyme treated DBG diets at a concentration of 1 gm/kg diet. ^c^FDBG (fermented dried brewer grains). ^d^DBG (dried brewer grains). ^e^Vitamin premix supplied per kilogram of diet: vitamin A, 10,000 IU; vitamin D3, 2000 IU; vitamin E, 6500 IU; vitamin K3, 1 mg; vitamin B1, 2560 mg; vitamin B2, 5000 mg; vitamin B6, 1500 mg; B5, 8 mg; niacin, 20000 mg; biotin, 0.25 mg; folic acid, 1000 mg; vitamin B12, 60 mg; Cu, 8 mg; Fe, 80 mg; Mn, 60 mg; Zn, 40 mg; Se, 0.15 mg

**Table 7:** The ingredients and nutrient level of diets during grower-finisher stage

Diet composition (%)	Experimental diets^a^							
	NC	PC^b^	FDBG 5%	FDBG 10%	FDBG 15%	DBG5% +Enz^b^	DBG10% +Enz^b^	DBG15% +Enz^b^
Yellow corn	62.25	62.25	60.20	57.90	55.60	59.30	56.45	53.40
Soybean meal	26.00	26.00	23.00	20.25	17.50	23.50	21.00	18.70
Corn gluten	3.90	3.90	3.90	3.90	3.90	3.90	3.90	3.90
FDBG ^c^	0	0	5.00	10.00	15.00	0	0	0
DBG ^d^	0	0	0	0	0	5.00	10.00	15.00
Soybean oil	3.60	3.60	3.60	3.60	3.60	4.00	4.30	4.60
Calcium carbonate	1.00	1.00	1.00	1.00	1.00	1.00	1.00	1.00
Calcium diphasic phosphate	1.30	1.30	1.30	1.30	1.30	1.30	1.30	1.30
Common salt	0.30	0.30	0.30	0.30	0.30	0.30	0.30	0.30
Premix ^e^	0.90	0.90	0.9	0.90	0.90	0.90	0.90	0.90
L-Lysine	0.25	0.25	0.3	0.35	0.4	0.3	0.35	0.40
DL-Methionine	0.20	0.20	0.20	0.20	0.20	0.20	0.20	0.20
Choline chloride	0.20	0.20	0.20	0.20	0.20	0.20	0.20	0.20
Anti-mycotoxin	0.10	0.10	0.1	0.1	0.1	0.1	0.1	0.1
Calculated composition								
ME (Kcal/Kg)	3200	3200	3201	3201	3200	3203	3202	3200
CP (%)	20.5	20.5	20.5	20.50	20.50	20.50	20.50	20.50
EE %	6.15	6.15	6.38	6.62	6.85	6.76	7.16	7.61
CF (%)	2.46	2.46	2.84	3.23	3.61	3.02	3.58	4.14
Calcium (%)	1.05	1.05	1.04	1.03	1.03	1.04	1.04	1.03
Available phosphorous (%)	0.45	0.45	0.43	0.41	0.39	0.43	0.41	0.39
Lysine (%)	1.16	1.16	1.16	1.16	1.17	1.16	1.16	1.17
Methionine (%)	0.56	0.56	0.56	0.57	0.57	0.56	0.57	0.57

^a^NC (negative control), PC (positive control), FDBG5% (5% fermented dried brewers grains), FDBG10% (10% fermented dried brewer grain), FDBG15% (15% fermented dried brewers grains), DBG5%+Enz (5%dried brewers grains mixed with enzymes), DBG10%+Enz (10% dried brewers grains mixed with enzymes), DBG15%+Enz (15% dried brewers grains mixed with enzymes). ^b^Commercial multi-exogenous enzyme was added was added to PC and enzyme treated DBG diets at a concentration of 1 gm/kg diet. ^c^FDBG (fermented dried brewer grains). ^d^DBG (dried brewer grains). ^e^Vitamin premix supplied per kilogram of diet: vitamin A, 10,000 IU; vitamin D3, 2000 IU; vitamin E, 6500 IU; vitamin K3, 1 mg; vitamin B1, 2560 mg; vitamin B2, 5000 mg; vitamin B6, 1500 mg; B5, 8 mg; niacin, 20000 mg; biotin, 0.25 mg; folic acid, 1000 mg; vitamin B12, 60 mg; Cu, 8 mg; Fe, 80 mg; Mn, 60 mg; Zn, 40 mg; Se, 0.15 mg

### Samples collection

At the end of experimental period. The birds euthanized by cervical dislocation and the blood samples (n = 5 per replicate) were collected in sterilized tubes from the brachial vein and the serum was separated for biochemical analyses. Following blood collection, these birds were manually defeathered and eviscerated to calculate the carcass weight together with the weights of gizzard, liver, spleen, thymus, bursa and abdominal fat. Samples (n = 5 per replicate) from breast and thigh meat were quickly obtained and stored at -20ºC until analysis of moisture, protein, and cholesterol in breast and cholesterol content in thigh. Small pieces (n = 5 per replicate), approximately 2 cm in the middle of proventriculus and pancreas and 4 cm of duodenum (distal loop) were excised, flushed with phosphate buffer saline, collected into Eppendorf cap lock tube and stored in -80ºC for subsequent RNA extraction.

### Measurement of growth performance and carcass traits:

Birds and feed intake in each pen were weighed weekly and body weight gain (**BWG**), feed intake (**FI**), and feed conversion ratio (**FCR**) were calculated. At the end of experimental period BWG, FI and FCR were calculated for total growing period (days 1–38). The carcass weight (dressing yield) was calculated as the percentage of live weight and weight of gizzard, liver, spleen, thymus, bursa and abdominal fat was calculated as a percentage of carcass weight.

### RNA Extraction, Reverse Transcription, and Quantitative Real-Time PCR

For each sample, total RNA was extracted from the proventriculus, pancreas and duodenum by RNeasy Mini Kit (Qiagen, Cat. No. 74,104) according to the manufacturer’s directions. The quantity and purity of total RNA was determined using a NanoDrop (ND-8000 spectrophotometer, Thermo Fisher Scientific, Waltham, USA). The isolated RNA of each sample was reverse transcribed using RevertAid™ H Minus kits (Fermentas Life Science, Pittsburgh, PA, USA). The cDNA (One µL) was mixed with 2x SYBR® Green PCR mix (12.5 µL), and RNase free water (5.5 µL), then 0.5 µL of each forward and reverse primer for the selected genes were added. The primers’ sequences of selected digestive and nutrient transporters genes were designed as previously described in Kheravii et al. [55], Table 8. The GAPDH was used as an internal control to normalize target gene expression levels [94]. The real-time PCR amplification was done with Rotor-Gene Q2 plex (Qiagen Inc., Valencia, CA, USA).

**Table 8:** Primers Sequences and target genes used for quantitative real-time PCR

Genes^a^	Gene full name	Primer sequence (5′-3′)	Accession No.
PGA5	Pepsinogen A	F-TCCGTCTACCTGAGCAAGGAT R- AAGCAGGCGACGTACTTGTT	NM_204878.1
PGC	Pepsinogen C	F-ATCGGGATTGAGGA^↓^CTTCGC R- TGAAGACCTGGTTGGGAACG	NM_204877.2
AMY2A	Pancreatic alpha 2A amylase	F-CGGAGTG^↓^GATGTTAACGACTGG R-ATGTTCGCAGACCCAGTCATTG	NM_001001473.2
PNLIP	Pancreatic lipase	F-GCATCTGGGAAG^↓^GAACTAGGG R- TGAACCACAAGCATAGCCCA	NM_001277382.1
CCK	Cholecystokinin	F-AGGTTCCACTGGGAGGTTCT R-CGCCTGCTGTTCTTTAGGAG	XM_015281332.1
CELA1	Chymotrypsin-like elastase family, member 1	F-AGCGTAAGGAAATGGGGTGG R-GTGGAGACCCCATGCAAGTC	XM_015300368.1
GLUT1	Glucose transporter-1 (SLC2A1)	F-TCCTCCTGATCAACCGCAAT R-TGTGCCCCGGAGCTTCT	NM_205209.1
GLUT2	Glucose transporter-2 (SLC2A2)	F-TGATCGTGGCACTGATGGTT R-CCACCAGGAAGAC^↓^GGAGATA	NM_207178.1
CAT1	Cationic amino acid transporter-1 (SLC7A1)	F-CAAGAGGAAAACTCCAGTAATTGCA R- AAGTCGAAGAGGAAGGCCATAA	XM_015277945.1
CAT2	Cationic amino acid transporter-2 (SLC7A2)	F-TGCTCGCGTTCCCAAGA R- GGCCCACAGTTCACCAACAG	XM_015285435.1
LAT1	L-type amino acid transporter-1 (SLC7A7)	F-GATTGCAACGGGTGATGTGA R- CCCCACACCCACTTTTGTTT	KT876067.1
PepT1	Peptide transporter-1 (SLC15A1)	F-TACGCATACTGTCACCATCA R-TCCTGAGAACGGACTGTAAT	AY029615.1
PepT2	Peptide transporter-2 (SLC15A2)	F-TGACTGGGCATCGGAACAA R-ACCCGTGTCACCATTTTAACCT	NM_001319028.1
GAPDH	Glyceraldahyde -3-phosphate dehydrogenase	F-GGTGGTGCTAAGCGTGTTA R-CCCTCCACAATGCCAA	NM205518

^a^The genes analyzed in the tissues are listed as follow: PGA5 and, PGC in proventriculus; AMY2A, CCK1R, CCK, CELA1, PNLIP, in pancreas; and, GLUT1, GLUT2, CAT1, CAT2, LAT1, PepT1, PepT2 in duodenum

### Biochemical analyses

The serum biochemical indices: total plasma proteins, albumin, globulin, total cholesterol, triglycerides, high density conjugated protein (HDL), low density protein (LDL), alanine aminotransferase (ALT) and aspartate amino transferase (AST) were measured using diagnostic kits (Spinreact, Santa Coloma, Spain).

#### Analysis of cholesterol content in breast and thigh meat

The moisture and protein content of breast were calculated according to AOAC [92]. The total cholesterol in breast and thigh meat was determined by gas chromatography, as previously reported by Allain et al. [95].

### Economic analysis

The economic efficiency of substituting corn-soybean by fermented or enzyme-treated DBG was calculated from the input-output analysis (as per the prevailing value of the experimental diets and also the broiler body weight during the experimental period) as follows:

Total feed cost = total feed intake per bird × cost of one kg diet [96].

Feed cost/kg BW gain = feed conversion × cost of one kg diet [97].

Total expenses = total feed cost + average fixed costs [2].

Total revenue = live body weight × price/kg [98].

Gross margin = total revenue – total expenses [98].

Benefit-cost ratio = gross margin/total feed cost [99].

### Statistical analyses

The data were analyzed using GLM procedure of SPSS, after confirming the homogeneity among experimental groups using Levene’s test and normality using Shapiro-Wilk’s test. Tukey’s post hoc was used to test for significant differences between the mean values. Variation in the data was expressed as standard error of the mean (SEM) and the significance was set at 0.05. Relative fold changes in the expression of target genes calculated by the 2^−ΔΔCt^ method as described by Livak and Schmittgen [100].

## Abbreviations

DBG: Dried brewer grains
FDBG: Fermented dried brewer grains
NC: Negative control
PC: Positive control
Enz: Enzyme
PGC: Pepsinogen C
PGA5: Pepsinogen A
AMY2A: Pancreatic alpha 2A amylase
PNLIP: Pancreatic lipase
CELA1: Chymotrypsin-like elastase family, member 1
CCK: Cholecystokinin
CAT1: Cationic amino acid transporter-1 (SLC7A1)
CAT2: Cationic amino acid transporter-1 (SLC7A2)
GLUT1: Glucose transporter-1 (SLC2A1)
GLUT2: Glucose transporter-2 (SLC2A2)
LAT1: L-type amino acid transporter-1
Pep1: Peptide transporter-1 (SLC15A1)
Pep2: Peptide transporter-2 (SLC15A2)
GAPDH: Glyceraldahyde − 3-phosphate dehydrogenase
ANF: Antinutritional factors
NSP: Non-starch polysaccharides
LAB: Lactic acid bacteria
BWG: Body weight gain
FI: Feed intake
FCR: Feed conversion ratio
HDL: High density conjugated protein
LDL: Low density protein
ALT: Alanine aminotransferase and
AST: Aspartate amino transferase
GLM: General linear method
SEM: Standard error of the mean
AA: Amino acids

## Availability of data and materials

The datasets used and/or analysed during the current study are available from the corresponding author on reasonable request.
